# Comparison of Adult Hippocampal Neurogenesis and Susceptibility to Treadmill Exercise in Nine Mouse Strains

**DOI:** 10.1155/2017/5863258

**Published:** 2017-12-17

**Authors:** Jong Whi Kim, Sung Min Nam, Dae Young Yoo, Hyo Young Jung, Il Yong Kim, In Koo Hwang, Je Kyung Seong, Yeo Sung Yoon

**Affiliations:** ^1^Department of Anatomy and Cell Biology, College of Veterinary Medicine, Research Institute for Veterinary Science, Seoul National University, Seoul 08826, Republic of Korea; ^2^Department of Anatomy, College of Veterinary Medicine, Konkuk University, Seoul 05030, Republic of Korea; ^3^KMPC (Korea Mouse Phenotyping Center), Seoul National University, Seoul 08826, Republic of Korea

## Abstract

The genetic background of mice has various influences on the efficacy of physical exercise, as well as adult neurogenesis in the hippocampus. In this study, we investigated the basal level of hippocampal neurogenesis, as well as the effects of treadmill exercise on adult hippocampal neurogenesis in 9 mouse strains: 8 very commonly used laboratory inbred mouse strains (C57BL/6, BALB/c, A/J, C3H/HeJ, DBA/1, DBA/2, 129/SvJ, and FVB) and 1 outbred mouse strain (ICR). All 9 strains showed diverse basal levels of cell proliferation, neuroblast differentiation, and integration into granule cells in the sedentary group. C57BL/6 mice showed the highest levels of cell proliferation, neuroblast differentiation, and integration into granule cells at basal levels, and the DBA/2 mice showed the lowest levels. The efficacy of integration into granule cells was maximal in ICR mice. Treadmill exercise increased adult hippocampal neurogenesis in all 9 mouse strains. These results suggest that the genetic background of mice affects hippocampal neurogenesis and C57BL/6 mice are the most useful strain to assess basal levels of cell proliferation and neuroblast differentiation, but not maturation into granule cells. In addition, the DBA/2 strain is not suitable for studying hippocampal neurogenesis.

## 1. Introduction

Adult neurogenesis is a transient process for generating new neurons in the adult mammalian brain, which arise from the subgranular zone of the dentate gyrus and the subventricular zone of the lateral ventricles throughout adult life. Newly generated neural stem cells in the dentate gyrus pass through maturation stages, and the surviving neuroblasts migrate into the granular cell layer (GCL), where they finally become mature neurons [1–4]. Numerous studies have been conducted in adult hippocampal neurogenesis (AHN), including investigations into the pool of neural stem cells, the effects of neurotrophins, signaling pathways associated with AHN, the relationship of specific target genes with neural stem cells, and external conditions influencing AHN.

Major extrinsic factors influencing AHN are environmental enrichment, dietary moderation, antidepressant drugs, and exercise conditions [5, 6]. Physical exercise in particular has been shown to procure benefits for learning and memory and has also been shown to enhance long-term potentiation and AHN [7]. In addition, exercise training increases the size of the hippocampus and improves memory function in mice and aged humans [8, 9]. Exercise also has neuroprotective and therapeutic effects on neurodegenerative diseases, such as Parkinson's disease [10, 11], Huntington's disease [12], and Alzheimer's disease [13]. However, these studies have investigated AHN using a single inbred mouse strain or neurodegenerative disease models.

Strain-dependent genotypes and phenotypes from genetic background have been studied for decades. Using a genome sequencing approach, large differences in genome sequences were found among 17 inbred mouse strains, which could influence phenotypes, gene regulation, and functional variants [14]. In a study of genetic influence on hippocampal neurogenesis, the level of cell proliferation of neural progenitor cells in the subgranular zone of the dentate gyrus was different in 4 mouse strains: C57BL/6, BALB/c, CD1, and 129/SvJ. In another study, an analysis of 4 mouse strains, *Mus spretus*, A/J, C3H/HeJ, and DBA/2 J, found that the inherent genetic background determined the basal level of AHN and the composition cell type [3, 15]. Furthermore, the 129/SvEms, 129.SvJ, C57, and C3H strains all showed different susceptibilities to kainic acid administration [16]. In addition, inbred strains performed differently on memory tasks, such as the Morris water maze and the contextual fear conditioning test [17]. More specifically, the 129S6/SvEv, 129T2/SvEmsJ, C57BL/6, and C57BL/10 strains performed well on the memory tests, whereas the BALB/c strain exhibited intermediate performance [17]. The DBA/2 strain, on the other hand, performed poorly, which is likely due to an impairment in hippocampal function, regardless of visual acuity [17]. Thus, differences in the background genes of mouse strains result in diverse phenotypes in hippocampus-dependent behavior and AHN [18, 19].

Among inbred mouse strains, markers for cell proliferation and neuroblast differentiation in AHN are expressed differentially in the C57BL/6, ICR, and BALB/c mouse strains [20], and genetic differences influence the population of neural stem cells in a strain-dependent manner. In our previous study, we showed that the C57BL/6 strain showed a high susceptibility to a high-fat diet as well as body weight gain and a significant reduction in cell proliferation and neuroblast differentiation, whereas the C3H/He strain is relatively resistant to a high-fat diet and body weight gain [21]. There is no established grade list, however, of the pool of neural stem cells and AHN in commonly used inbred strains, such as 129/SvJ, C57BL/6, BALB/c, A/J, and other *Mus musculus* subspecies.

In the present study, we investigated the basal level of cell proliferation, neuroblast differentiation, and cell survival in eight commonly used inbred mouse strains (C57BL/6, BALB/c, A/J, C3H/HeJ, DBA/1, DBA/2, 129/SvJ, and FVB) and one outbred mouse strain (ICR) to elucidate strain-specific differences in AHN. In addition, we also observed susceptibility to treadmill exercise and its enhancing effects on AHN in these 9 mouse strains to understand the phenotypic variation with genetic background and to determine the best mouse strain for use in AHN studies. Our data confirmed that there is diversity in the basal level of AHN in 9 different mouse strains, and also suggests avenues for future investigations into the mechanism of AHN, as well as the selection of proper mouse strains for genetically engineered mouse models.

## 2. Methods

### 2.1. Experimental Animals

Six-week-old male C57BL/6J, A/J, BALB/c, C3H/HeJ, FVB, 129/SvJ, DBA/1, DBA/2, and ICR mice were purchased from Japan SLC Inc. (Shizuoka, Japan). The animals were housed in a specific pathogen-free animal facility at 23°C with 60% humidity, a 12 h/12 h light/dark cycle, with ad libitum access to food and tap water. The handling and care of the animals conformed to guidelines established in compliance with current international laws and policies (NIH Guide for the Care and Use of Laboratory Animals, NIH Publication number 85-23, 1985, revised 1996) and were approved by the Institutional Animal Care and Use Committee (IACUC) of Seoul National University (SNU-120913-1-2). All experiments were conducted with an effort to minimize the number of animals used and the suffering caused by the procedures used in the study.

### 2.2. Exercise Condition

**Figure 1:**
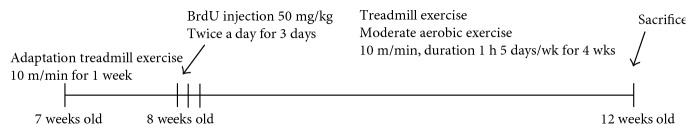
Experimental design of the present study. Treadmill exercise was adopted in 7-week-old mice for 1 week, and treadmill exercise was conducted for 4 weeks with 10 m/min speed for 1 h. During the first 3 days of exercise, BrdU was injected twice a day (at 8:00 AM and 8:00 PM) intraperitoneally to label newborn neural stem cells.

After a one-week acclimation to laboratory condition, each mouse strain was divided into 2 groups (*n* = 5 in each group): sedentary (SED) and exercise (EX) groups. The animals in the SED and EX groups were familiarized with treadmill exercise on a motorized treadmill (Model 1050 Exer3/6; Columbus Instruments, Columbus, OH, USA) for one week. In the EX group, running speed and durations were 10 m/min for 20 min on the first day, with an increase of 10 min/day until a total of 60 min/day was reached [22]. The animals in the SED group were placed on the treadmill without any running speed for the same period as the EX group. After familiarization, treadmill exercise was regularly practiced at 10 m/min for 60 min/day at 5 days/week for 4 weeks (Figure 1). This schedule was selected because a significant increase in proliferative activity and the production of new neurons in C57BL/6 and DBA/2 J mice has been seen after 28 days of running, while only a small (not significant) and transient increase in proliferative activity was seen in these strains after 42 days of running [23].

### 2.3. Measurement of Body Weight and Food Intake

Body weight was measured at 10:00 AM every week on Wednesday and at the end of the experiment. Food intake was measured and corrected for spillage by weighing the jars containing food every week between 9:00 and 10:00 AM. Food intake was calculated from the average intake during the 4-week experimental period and expressed as g/mouse/week. Body weight gain was calculated as the difference in body weight between 12 weeks and 8 weeks.

### 2.4. Labeling of Newly Generated Cells

To label newly generated cells in the hippocampus, intraperitoneal injections of 5-bromo-2′-deoxyuridine (BrdU, 50 mg/kg, Sigma-Aldrich, St. Louis, MO, USA) were given to all mice twice daily (8:00, 20:00) for three days at the start of the exercise (when the mice were at eight weeks of age). Animals were euthanized at 12 weeks of age, one day after the last exercise (Figure 1).

### 2.5. Tissue Preparation

Animals (*n* = 5 in each group) were anesthetized by an intraperitoneal injection of 1 g/kg urethane (Sigma-Aldrich) and perfused transcardially with 0.1 M phosphate-buffered saline (PBS, pH 7.4) followed by 4% paraformaldehyde in 0.1 M PBS. The brains were then dissected and postfixed in the same fixative for 12 h. The brain tissues were cryoprotected by infiltration with 30% sucrose overnight. Thirty-micrometer-thick brain sections were serially sectioned in the coronal plane using a cryostat (Leica, Wetzlar, Germany) and collected in six-well plates containing PBS at −20°C for further processing.

### 2.6. Immunohistochemistry

To obtain accurate data for immunohistochemistry, the free-floating sections from all animals were processed carefully under the same conditions. For each animal, tissue sections were selected from between 1.46 mm and 2.46 mm posterior to the bregma by referring to the mouse atlas by Franklin and Paxinos [24]. Ten sections, 90 *μ*m apart, were sequentially treated with 0.3% hydrogen peroxide (H_2_O_2_) in PBS for 30 min and 10% normal goat or rabbit serum in 0.05 M PBS for 30 min. They were then incubated with a rabbit anti-Ki67 antibody (1 : 1000; Abcam, Cambridge, UK) or goat anti-DCX antibody (1 : 50; Santa Cruz Biotechnology, Santa Cruz, CA, USA) overnight at 25°C and subsequently treated with either a biotinylated goat anti-rabbit IgG or a rabbit anti-goat IgG and a streptavidin-peroxidase complex (1 : 200, Vector Labs, Burlingame, CA, USA). Sections were visualized by reaction with 3,3′-diaminobenzidine tetrachloride (Sigma) in 0.1 M Tris-HCl buffer (pH 7.2) and dehydrated and mounted in Canada balsam (Kanto Chemical, Tokyo, Japan) onto gelatin-coated slides.

### 2.7. Immunofluorescence

For BrdU and NeuN double immunofluorescence, the sections were treated with 2 N HCl for 30 min at 37°C and were incubated with a mixture of mouse anti-NeuN (1 : 1000; Millipore, Temecula, CA, USA) and rat anti-BrdU (1 : 200; Abd Serotec, Bio-Rad Laboratories, Inc., Grand Island, NY, USA) for 2 h at 25°C, followed by overnight incubation at 4°C. After washing with PBS, the sections were subsequently incubated with secondary antibodies, FITC-conjugated goat anti-mouse IgG (1 : 100; Jackson ImmunoResearch, PA, USA), and Cy3-conjugated goat anti-rat IgG (1 : 100; Jackson ImmunoResearch, PA, USA), for 2 h. After that, the sections were mounted on silane-coated slides with DAPI-containing mounting medium (Vector Labs, CA, USA) for nuclei staining.

### 2.8. Microscopic Analysis

Two independent masked investigators counted Ki67-, DCX-, BrdU-, or BrdU/NeuN-labeled cells in the dentate gyrus at 400x magnification under a light microscope (BX51, Olympus, Tokyo, Japan). All Ki67-, DCX-, BrdU-, or BrdU/NeuN-labeled cells were counted bilaterally in 10 sections (90 *μ*m apart from each other) across the entire dentate gyrus between 1.46 mm and 2.46 mm posterior to the bregma by referring to the mouse atlas by Franklin and Paxinos [24].

### 2.9. Statistical Analysis

Statistical analysis was performed using SPSS V.20.1 (IBM Corporation, Armonk, NY, USA). Experimental groups were compared using two-way analysis of variance (ANOVA), followed by a least significant difference (LSD) post hoc analysis.

## 3. Results

### 3.1. Effects of Strain and Exercise on Body Weight and Food Intake

**Figure 2:**
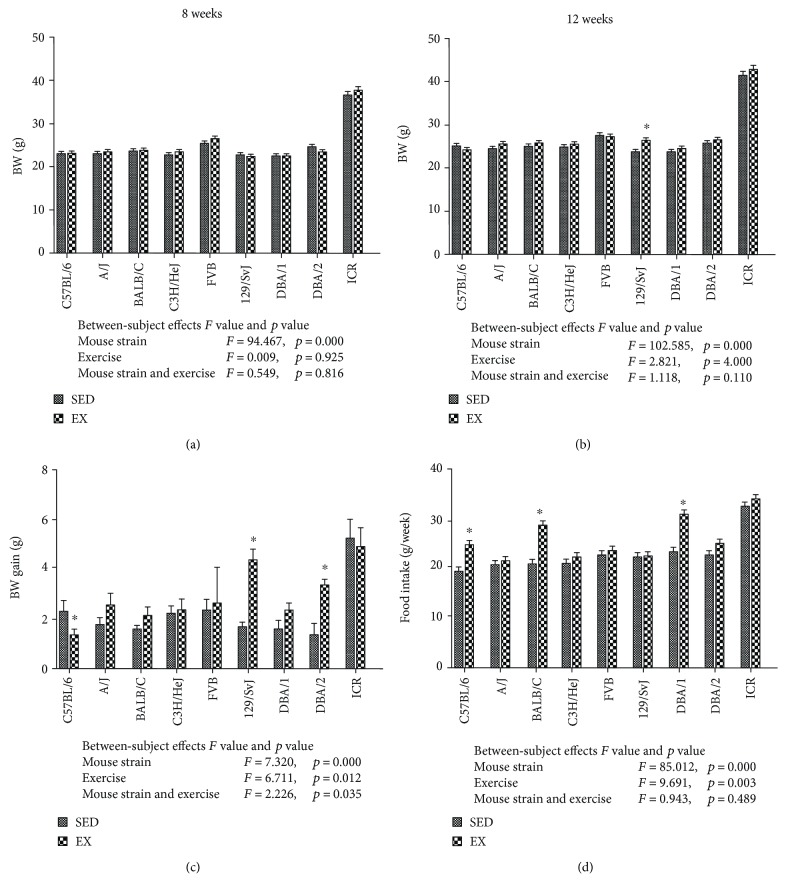
Body weight at the beginning (8 weeks of age) (a) and end (12 weeks of age) (b) of exercise and its control group (*n* = 5 per group). Body weight gains analysis was determined by calculating the difference in body weight at 12 weeks and 8 weeks (c). Food intake was calculated from the mean intake during the 4-week experimental period and expressed as g/mouse/week (d). *F* value and *p* value of between-subjects effects in mouse strain, exercise, and mouse strain and exercise showed in under the graph. ∗ indicates a significant difference between the sedentary and exercise groups (*p* < 0.05); data are shown as mean ± SEM.

At eight weeks of age, each inbred mouse strain showed similar body weight, except for the outbred ICR strain, which had a significantly higher body weight than the inbred mice (Figure 2(a)). The body weight of the animals tended to increase with age by 12 weeks in both the SED and EX groups, with only the EX group of the 129/SvJ strain showing a significant increase in body weight compared to the SED group of the same strain (Figures 2(a) and 2(b)). Statistical analysis was also performed between-subjects effects in mouse strain, exercise, and mouse strain and exercise. *F* value and *p* value showed in Figures 2(a) and 2(b). Between-subjects effects of mouse strains, and exercise showed significance, but mouse strain and exercise have no significance. Among the inbred strains, there were significant differences in body weight gain between the EX and SED groups in the C57BL/6, 129/SvJ, and DBA/2 strains. There was a significant reduction in body weight gain in the C57BL/6 strain at the age of 12 weeks after exercise, whereas a significant increase in body weight gain was observed in the EX group of the 129/SvJ and DBA/2 mouse strains, as compared to that of the SED group (Figure 2(c)). To confirm the correlation of body weight gain with food intake, we analyzed food intake in the SED and EX groups. Between-subjects effects of mouse strains, exercise, and mouse strain and exercise showed significant, *F* value and *p* value showed in Figures 2(c) and 2(d). Exercise influenced food intake in some mouse strains; food intake significantly increased in the EX group of the C57BL/6, BALB/c, and DBA/1 strains compared to those in the SED group (Figure 2(d)). In contrast, the C57BL/6 strain showed a reverse correlation between exercise and body weight gain, and BALB/c and DBA/1 mice showed no significant change in body weight or food intake (Figures 2(c) and 2(d)).

### 3.2. Effects of Strain and Exercise on Cell Proliferation

**Figure 3:**
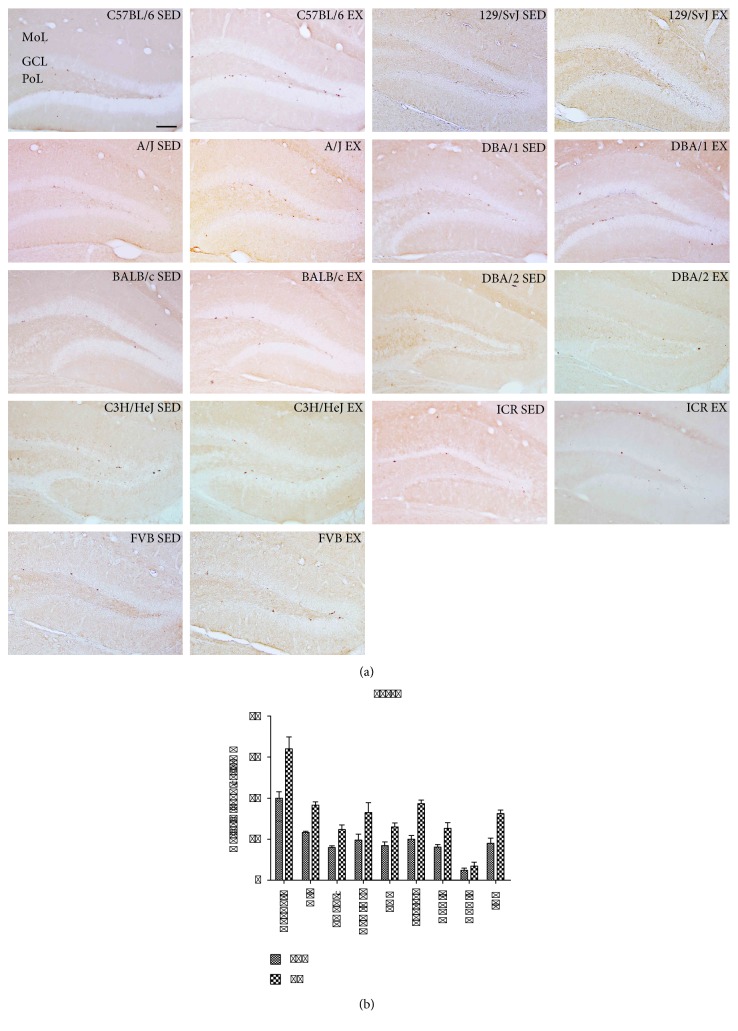
Immunohistochemistry for Ki67 in the dentate gyrus of sedentary and exercise mice of 9 different strains (a). GCL, granule cell layer; ML, molecular layer; PoL, polymorphic layer. Scale bar = 100 *μ*m. Quantitative analysis of Ki67-positive nuclei per section in sedentary and exercise mice (b) (*n* = 5 per group); ∗∗ indicates a significant difference between the sedentary and exercise groups (*p* < 0.01). Data are shown as mean ± SEM.

To observe the proliferation of hippocampal neural progenitor cells in the 9 mouse strains, as well as their susceptibility to the effects of 4 weeks of treadmill exercise, cells in the subgranular zone of the dentate gyrus were stained with the proliferation marker Ki67, and the mean number of Ki67 immunoreactive cells was calculated. In the SED groups, there were different population levels of Ki67-positive cells in the different mouse strains (Figure 3(b)). The number of Ki67-positive cells is shown in Table 1. Notably, the mean number of Ki67-positive cells was the highest in C57BL/6 mice (20.00 ± 1.58, Figures 3(a) and 3(b), Table 1) and the lowest in DBA/2 mice (2.40 ± 0.51, Figures 3(a) and 3(b), Table 1). Similar numbers of Ki67-positive cells in the dentate gyrus were observed in other strains, including the AJ, 129/SvJ, C3H, ICR, FVB, and BALB/c strains (Table 1).

Compared to the SED group, the number of Ki67-positive cells was significantly increased in the dentate gyrus of the EX group in all mouse strains except for the DBA/2 strain (Table 1). The increase in percentage of Ki67-positive cells in the EX group for each mouse strain is shown in Table 1. Proliferating cells were most prominently increased (186.00%) in the 129/SvJ strain after exercise and were least prominently increased (141.60%) in the DBA/2 strain. Across all strains, the average number of Ki67-positive cells in the dentate gyrus in the EX group was increased to 162.24% compared to those in the SED group (Table 1). *p* value in the Supplementary Table 1 obtained from LSD post hoc analysis was compared to the cross-matched group. Statistical analysis was also performed between-subjects effects in mouse strain, exercise, and mouse strain and exercise. Between-subjects effects of mouse strains, exercise, and mouse strain and exercise showed significant. *F* value and *p* value were showed in Supplementary Table 1.

### 3.3. Effects of Strain and Exercise on Neuroblast Differentiation

**Figure 4:**
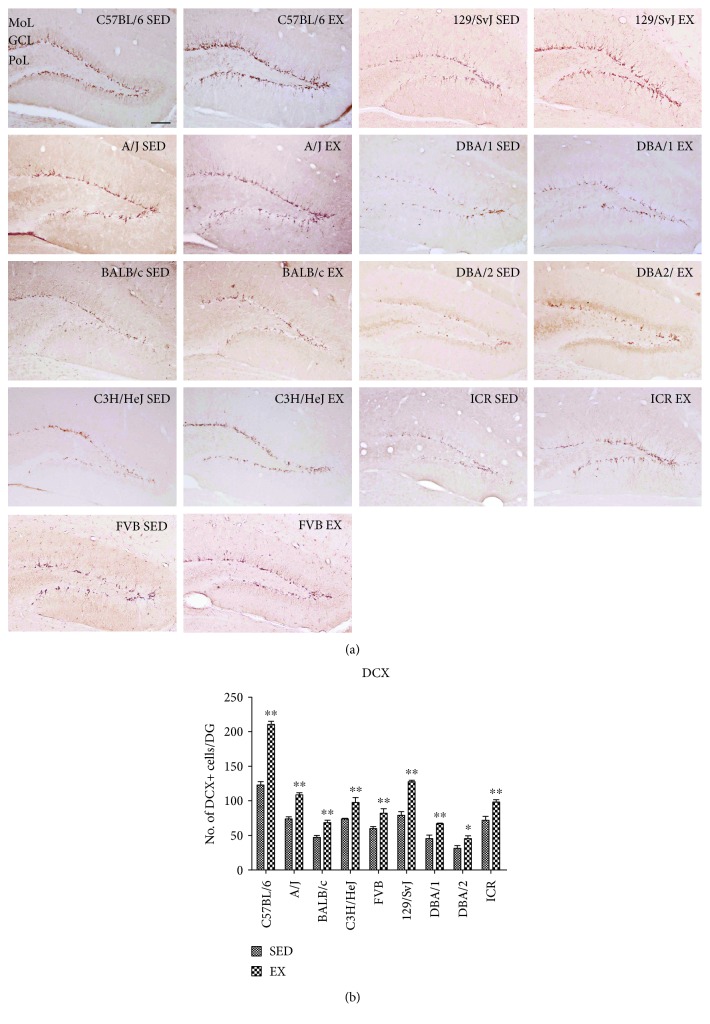
Immunohistochemistry for doublecortin (DCX) in the dentate gyrus of sedentary and exercise mice of 9 different strains (a). GCL, granule cell layer; ML, molecular layer; PoL, polymorphic layer. Scale bar = 100 *μ*m. Quantitative analysis of DCX-immunoreactive neuroblasts per section in sedentary and exercise mice (b) (*n* = 5 per group); ∗ indicates a significant difference between the sedentary and exercise groups (*p* < 0.05), and ∗∗ indicates a significant difference between the sedentary and exercised groups (*p* < 0.01). Data are shown as mean ± SEM.

Doublecortin (DCX) immunohistochemistry, which is a marker for differentiated neuroblasts found in the subgranular zone of the dentate gyrus, was used to examine the basal levels of neuroblast differentiation and the effects of 4 weeks of treadmill exercise on the differentiation of hippocampal neural progenitor cells. In the SED group, the mean number of DCX-immunoreactive neuroblasts was different in each mouse strain (Figures 4(a) and 4(b)). The mean number of DCX-immunoreactive neuroblasts was highest in the C57BL/6J strain (128.80 ± 4.86, Figures 4(a) and 4(b), Table 1) and lowest in the DBA/2 strain (31.00 ± 4.18, Figures 4(a) and 4(b), Table 1). We divided the animal strains into 2 groups: those with an intermediate number of DCX-immunoreactive neuroblasts and those with a lower number. The number of DCX-immunoreactive neuroblasts in the intermediate group was 60.00–79.20 cells and included the 129/SvJ, AJ, C3H, ICR, and FVB strains (Figure 4(b) and Table 1). In contrast, the BALB/c, DBA/1, and DBA/2 strains had 45.40–47.20 DCX-immunoreactive neuroblasts and were included in the lower group (Figure 4(b) and Table 1).

Exercise significantly increased the number of DCX-immunoreactive neuroblasts in the dentate gyrus of all mouse strains compared to that of the respective strains in the SED group, as shown in Table 1. The increase in the number of DCX-immunoreactive neuroblasts was the most prominent (171.17% increase) in the C57BL/6 strain and the lowest (132.43%) in the C3H/HeJ strain (Table 1). *p* value in the Supplementary Table 2 obtained from LSD post hoc analysis was compared to the cross-matched group. Statistical analysis was also performed between-subjects effects in mouse strain, exercise, and mouse strain and exercise. Between-subjects effects of mouse strains, exercise, and mouse strain and exercise showed significance. *F* value and *p* value were showed in Supplementary Table 2.

### 3.4. Effects of Strain and Exercise on Integration into Mature Granule Cells

**Figure 5:**
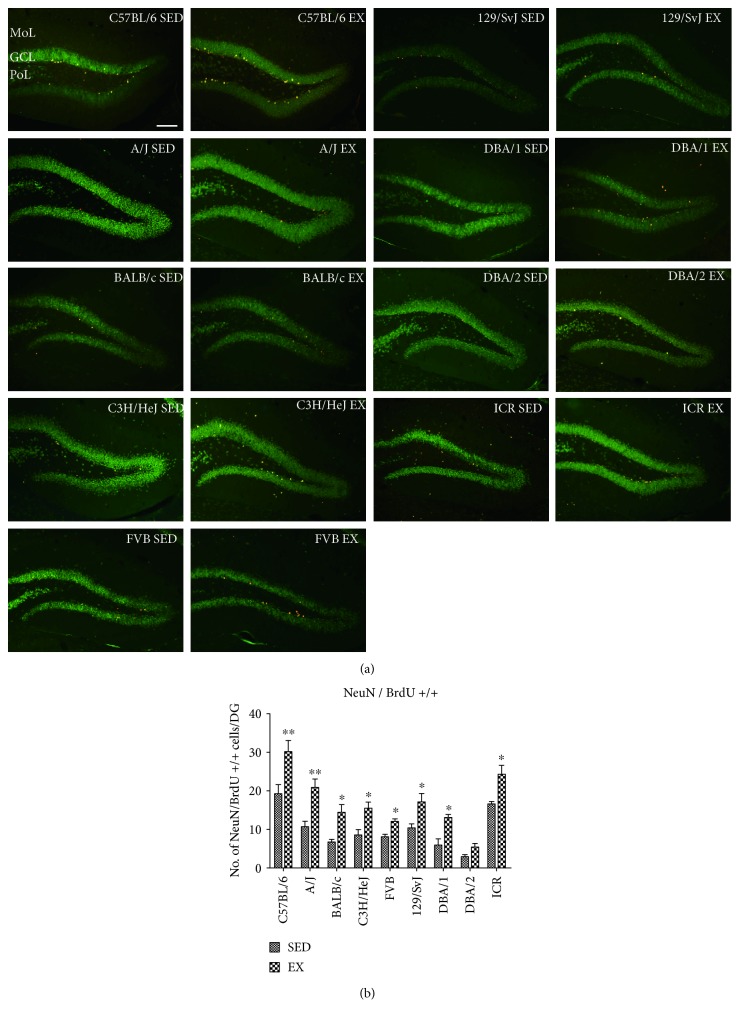
Double immunofluorescence staining for BrdU (red) and NeuN (green) in the dentate gyrus of sedentary and exercise mice of 9 different strains (a). GCL, granule cell layer; ML, molecular layer; PoL, polymorphic layer. Scale bar = 100 *μ*m. Quantitative analysis of BrdU and NeuN double-labeled cells per section in sedentary and exercise mice (a) (*n* = 5 per group); quantitative cell number of BrdU (red) and NeuN (green) double-positive cells (b); ∗ indicates a significant difference between the sedentary and exercise groups (*p* < 0.05), and ∗∗ indicates a significant difference between the sedentary and exercise groups (*p* < 0.01). Data are shown as mean ± SEM.

In this study, we quantified BrdU and NeuN double-positive cells in the GCL to evaluate integration into mature granule cells in the dentate gyrus, and the mean number of NeuN and BrdU double-positive cells was calculated to compare the effects of strain and exercise on neurogenesis. In the SED group, BrdU and NeuN double-positive cells were the most abundant in the C57BL6 strain (21.20 ± 1.93, Figure 5(a) and Table 1) compared to other mouse strains and were the least abundant in the DBA/2 strain (2.20 ± 0.37, Figure 5(a) and Table 1). The ICR strain also had a high number of BrdU and NeuN double-positive cells in the dentate gyrus, while other strains had an intermediate number of BrdU and NeuN double-positive cells (Figures 5(a) and 5(b), Table 1). In the EX group, the number of NeuN/BrdU double-positive cells was significantly increased compared to those in the SED group in all 9 mouse strains, (Table 1). The average number of NeuN/BrdU double-positive cells across all mouse strains was 160.39%, and the increase in NeuN/BrdU double-positive cells was most prominent in the DBA/1 and A/J strains and least prominent in the C57BL/6 and ICR mice (Table 1).

The ratio of BrdU and NeuN double-positive cells and DCX-immunoreactive neuroblasts (BrdU + NeuN/DCX) was also calculated and is shown in Table 1. The ratio of BrdU + NeuN/DCX was highest (17.70%) in the ICR mice and lowest (7.80%) in the DBA/2 mice. In the EX group, the ratio of BrdU + NeuN/DCX was most prominent (18.78%) in the ICR mice, and least prominent (8.62%) in the DBA/2 mice (Table 1). The other mouse strains did not show any significant differences in this ratio between groups (Table 1). *p* value in the Supplementary Table 3 obtained from LSD post hoc analysis was compared to the cross-matched group. Statistical analysis was also performed between-subjects effects in mouse strain, exercise, and mouse strain and exercise. Between-subjects effects of mouse strains, exercise showed significant and exercise, and mouse strain and exercise have no significant. *F* value and *p* value were showed in Supplementary Table 3.

## 4. Discussion

Our basic objectives were to investigate the differences in cell proliferation, neuroblast differentiation, and integration into mature granule cells in the dentate gyrus and evaluate the efficacy of treadmill exercise on the population of AHN in 9 mouse strains. In this study, we selected 9 mouse strains that are widely used in biomedical research, as well as mouse family tree groups, which were classified with informative single nucleotide polymorphism markers sorted by genetic relationship, resulting in the organization of 102 strains into 7 family tree groups [25]. On the basis of this report, the A/J, BALB/c, and C3H/HeJ strains were included in group 1, the FVB strain in group 2, the C57BL/6J strain in group 4, the 129X1/SvJ strain in group 5, and the DBA strains in group 6 [25]. We observed that the level of cell proliferation and neuroblast differentiation in normal mice was C57BL/6 > (A/J = 129X1/SvJ) > C3H/HeJ > ICR > FVB > BALB/c > DBA/1 > DBA/2. In addition, the efficacy of integration into mature neurons was C57BL/6 > ICR > (A/J = 129X1/SvJ) > C3H/HeJ > FVB > BALB/c > DBA/1 > DBA/2. These results suggest that the potential for neurogenesis is most prominent in the C57BL/6 and ICR strains and are the lowest in the DBA/2 strain. These results are consistent with previous reports that precursor cell proliferation and net production of new neurons is strikingly higher in C57BL/6 strains [26–28]. On the other hand, we found that the DBA/1 and DBA/2 strains have a low capacity of cell proliferation and neuroblast differentiation in the dentate gyrus.

In the present study, ICR mice had a higher ratio of BrdU + NeuN/DCX positive cells than other strains. This result suggests that there is a higher efficacy for the integration of neuroblasts into mature neurons in ICR mice as compared to the other 8 inbred mouse strains. This result was consistent with a previous study that showed that proliferation was highest in the C57BL/6 strain, and the survival rate of newborn cells was highest in the ICR strain [3]. Other studies reported lower cell proliferation in FVB/NJ, ICR, and BALB/c mice compared to that in C57BL/6J strain [20, 29], with no differences in the number of surviving cells between C57BL/6J and FVB/NJ mice [29]. In addition, DCX-immunoreactive neuroblasts were more abundant in the C57BL/6 strain than in ICR and BALB/c mice [20]. However, they did not observe any significant differences in the number of Ki67- and DCX-immunoreactive cells between BALB/c and ICR mice [20]. This discrepancy with our data may be associated with differences in the experimental paradigm and the tissue processing methods that were used. In this study, we used our animals at 12 weeks of age and sectioned the tissue at 30 *μ*m thickness, while previous study used mice at 8 weeks of age and also sectioned with a thickness of 5 *μ*m and used paraffin embedding [20].

Several lines of evidence show that there are strain-dependent differences in various phenotypes, including hippocampus-dependent learning and memory and long-term potentiation (LTP) in the hippocampus. Electrophysiological and behavioral tests showed strain-dependent differences in LTP and hippocampal-dependent learning memory in C57BL/6, CBA/J, DBA/2J, and 129SvEms mice [30]. In particular, C57BL/6 mice exhibited long-lasting LPT, higher behavioral scores, and better long-term memory, while DBA/2J and CBA/J mice exhibited deficient long-term memory [30]. In the behavioral task in response to stimulation, phenotypic differences were observed across inbred mouse strains and was especially notable in the behavioral phenotypes of C57BL/6 and DBA/2 mice. In the open-field test and the Morris water maze task, C57BL/6 mice performed better than other inbred strains, and DBA/2 performed worse [30]. Other C57 substrains, such as C57BL/10, C57BR, and C57L, showed higher levels of spatial memory, longer-lasting LTP, and reduced anxiety behavior [30]. The observed behavioral and electrophysiological superiorities of C57BL/6 mice and the inferiorities of DBA/2 mice [30] are consistent with our data. Furthermore, our results showed that C57BL/6 mice have the largest NSC population and NSC lineage, while DBA/2 have a small NSC population and NSC lineage. Differences in AHN phenotype in 9 mouse strains are related to genuinely inherent genetic backgrounds, and differences in behavioral tests, LTP, and SNP markers may be correlated with our data in cell proliferation, differentiation, and survival.

There is evidence that newly generated neurons contribute to learning and memory function, synaptic formation, and integration into the hippocampal network circuit [31–33]. New neurons in the hippocampus make distinctive contributions to hippocampal function; at different stages of maturation, cells of NSC lineage also play unique roles in hippocampal function [34]. Many studies have shown that newborn neurons in the hippocampus play potential roles in pattern separation and the erasure of memories [35]. Current studies have also suggested that adult born dentate granule neurons are involved in pattern separation and the reactivation of dentate granule neurons [36]. This series of studies indicates that AHN and the associated newly generated neurons, along with integrated neurons, contribute to hippocampal learning function. The previously mentioned strain-dependent differences in performance on behavioral tests, LTP [30], and basal levels of AHN might regulate hippocampal function and determine differences in hippocampus phenotype across mouse strains. Several hippocampal genes related to neurological phenotypes were different in eight inbred mouse strains (A/J, Balb/cByJ, C3H/HeJ, C57BL/6J, DBA/2J, FVB/NJ, SJL/J, and 129S1/SvImJ) [36]. These differences in gene expression could be related to phenotypic differences in the hippocampus [36]. In addition, the C57BL/6 strain performed well on memory tasks, including the Morris water maze and the contextual fear conditioning test, whereas the DBA/2 strain performed poorly on both tests [17]. In our study, strain-dependent differences in the level of AHN also suggest that there is an effect of genetic background, which may explain strain-dependent differences in memory function. Furthermore, the population differences of newborn and integrated neurons between 9 mouse strains may explain differences in the learning and memory functions of the hippocampus.

In the present study, we could not elucidate the factors that induce differences in neurogenesis among mouse strains. There has been a report, however, that pregnenolone sulfate (PREGS) level is an important factor in differences in neurogenesis that are observed among the strains. In the DBA/2 strain, PREGS levels are significantly lower than in the C57BL/6, BALB/c, ddY, and ICR strains, while dehydroepiandrosterone sulfate (DHEAS) concentrations in the DBA/2 strain were significantly higher than those in other strains [37]. PREGS is known to promote neurogenesis [38] and increase the survival of newly generated cells [39]. In addition, PREGS improves spatial cognitive performance, as well as ameliorates the reduction in the survival and maturation of newborn neuronal cells in a mouse model of Alzheimer's disease [40]. In contrast, DHEAS decreases activated Akt levels and increases apoptosis [41].

Neurogenesis in the hippocampus is regulated at each level of cell proliferation, neuroblast differentiation, and survival. Regulation of neurogenesis has been controlled in many paradigms, including physical exercise [8, 42]. Physical exercise enhances AHN and improves learning and memory and long-term potentiation [43]. In the present study, we observed that physical exercise significantly increased cell proliferation, neuroblast differentiation, and integration into mature neurons in 8 mouse strains, but not in the DBA/2 strain. This result is consistent with our previous studies in which we showed that physical exercise increases neurogenesis and the number of dendrites of DCX-immunoreactive neuroblasts in the dentate gyrus [44–46]. The responsiveness to physical exercise was most prominent in the BALB/c strain and least prominent in the C57BL/6 strain. This result is consistent with a previous study that showed that running for 43 days increases neurogenesis to the greatest extent in BALB/cByJ mice and to the least extent in C57BL/6 mice [28].

The mechanism by which exercise increases AHN may be explained by systemic improvement and changes in neurotrophins, signaling pathway-related ligands, and receptors. Exercise increases the growth of blood vessels in the hippocampus and blood flow in the dentate gyrus of the hippocampus [8, 47]. In addition, exercise training increases the size of the hippocampus and improves memory function and also reduces normal shrinking of the hippocampal region in aging humans [9]. Some investigations provide clues about androgenic mediation of neurogenesis; mild exercise increases neurogenesis through an increase in androgenic enzyme and androgenic receptor in the hippocampus [48]. Exercise increases the production and secretion of brain-derived neurotrophic factor (BDNF) and mRNA expression of its receptor, tyrosine kinase (TrkB), in the hippocampus [49–52]. Inhibiting the action of BDNF with TrkB-IgG blocks the effect of exercise on downstream pathways regulated by BDNF that are important for synaptic plasticity, including cAMP response elements binding protein (CREB) and synapsin I [50]. Other studies have also shown that physical exercise increases neurogenesis by the induction of insulin-like growth factor-1 and vascular endothelial growth factor [53, 54] and also has beneficial effects on LTP by altering *N*-methyl-d-aspartate subunit contribution [55].

In conclusion, we showed that there are basal strain-dependent differences in AHN, as well as differences in the effectiveness of physical exercise on AHN in 9 mouse strains. In normal mice, AHN was the most abundant in C57BL/6 mice and was the least abundant in DBA/2 mice. However, integration into mature neurons was most effective in ICR mice. The responsiveness to physical exercise was most prominent in the BALB/c strain, and least prominent in the C57BL/6 strain. Choosing the correct inbred mouse strain for transgenic or knockout mouse models for common neurological studies requires significant knowledge of the origin of phenotype for each inbred mouse strain. Furthermore, knowledge about the differences in phenotype between inbred mouse strains may contribute to our understanding of strain-dependent genetic influences on AHN and may therefore aid in choosing the correct approach for generating a suitable animal disease model. Our data showed varying degrees of basal AHN level in 9 mouse strains. Therefore, in the design of AHN studies, it is necessary to take into account the genetic differences related to AHN in each mouse strain. Our study also indicates that the level of physical activity in the study, such as treadmill exercise, should be taken into consideration. Current approaches to reveal the mechanism of AHN use reverse genetics, but the elucidation of clear mechanisms for differences in AHN requires a forward genetics approach with different mouse strains. These results provide information for the selection of appropriate mouse strains and the ideal conditions for AHN experiments in the field of neuroscience.

## Figures and Tables

**Table 1:** Summary of NSC proliferation, differentiation, and number of mature neurons across mouse strains, and efficacy of integration into mature neurons from proliferating NSCs and differentiating neuroblasts. (*n* = 5 per group); ∗ indicates a significant difference between the sedentary and exercise groups (*p* < 0.05), and ∗∗ indicates a significant difference between the sedentary and exercise groups (*p* < 0.01). Data are shown as mean ± SEM. NSC: neural stem cells.

	Strains	CB7BL/6J		A/J		BALB/c		C3H/HeJ		FVB		129/SvJ		DBA/1		DBA/2		ICR		Total increase %
		Mean	SE	Mean	SE	Mean	SE	Mean	SE	Mean	SE	Mean	SE	Mean	SE	Mean	SE	Mean	SE	
Ki-67	SED	20.00	± 1.58	11.60	± 0.24	8.00	± 0.32	9.80	± 1.46	8.40	± 0.93	10.00	± 0.89	8.00	± 0.71	2.40	± 0.51	9.00	± 1.22	
Proliferation	EX	32.20	± 2.97	18.20	± 0.86	12.40	± 1.03	16.40	± 2.48	13.00	± 0.95	18.60	± 0.93	12.60	± 1.44	3.40	± 0.98	16.20	± 0.86	
	Increase %	161.00	^∗∗^	156.90	^∗∗^	155.00	^∗∗^	167.35	^∗∗^	154.76	^∗∗^	186.00	^∗∗^	157.50	^∗∗^	141.67	ns	180.00	^∗∗^	162.24
DCX	SED	122.80	± 4.86	73.60	± 2.91	47.20	± 2.40	73.40	± 1.12	60.00	± 2.77	79.20	± 5.34	45.40	± 4.79	31.00	± 4.18	71.60	± 5.91	
Differentiation	EX	210.2	± 4.89	108.6	± 2.94	68.4	± 3.49	97.2	± 7.44	82.2	± 6.26	127.2	± 2.48	73.00	± 1.10	45.20	± 4.31	97.8	± 3.62	
	Increase %	171.17	^∗∗^	147.55	^∗∗^	144.92	^∗∗^	132.43	^∗∗^	137.00	^∗∗^	160.61	^∗∗^	160.79	^∗∗^	145.81	^∗^	136.59	^∗∗^	148.54
BrdU/NeuN +/+	SED	21.20	± 1.81	8.00	± 1.05	5.00	± 0.55	6.40	± 1.03	6.00	± 0.55	7.80	± 0.73	4.40	± 1.25	2.20	± 0.37	12.40	± 0.51	
Maturation & survival	EX	31.00	± 2.18	15.60	± 1.69	10.80	± 1.50	11.60	± 1.21	9.00	± 0.55	12.80	± 1.65	9.80	± 0.58	4.00	± 0.77	18.20	± 1.74	
	Increase %	146.23	^∗∗^	195.00	^∗∗^	216.00	^∗^	181.25	^∗^	150.00	^∗^	164.10	^∗^	222.73	^∗^	181.82	ns	146.77	^∗^	160.39

		CB7BL/6 J		A/J		Balb/c		C3H/HeJ		FVB		129/Svj		DBA/1		DBA/2		ICR	
		Mean	SE	Mean	SE	Mean	SE	Mean	SE	Mean	SE	Mean	SE	Mean	SE	Mean	SE	Mean	SE	

Efficacy of cell maturation	SED-BrdU + NeuN/Ki67	106.00	± 16.61	69.09	± 9.16	63.17	± 7.70	72.99	± 18.17	76.31	± 11.45	78.14	± 3.55	56.39	± 16.57	125.00	± 46.10	154.08	± 32.48	85.59
	EX-BrdU + NeuN/Ki67	96.27	± 13.39	85.32	± 7.87	91.78	± 16.63	75.12	± 11.02	70.51	± 5.91	69.09	± 8.97	80.87	± 7.48	136.43	± 20.40	113.38	± 11.96	88.56
	EX/SED %	90.82		123.49		145.28		102.93		92.40		88.42		143.41		109.14		73.59		108.65
	SED-BrdU + NeuN/DCX	17.26	± 1.34	11.04	± 1.65	10.60	± 0.98	8.70	± 1.39	9.96	± 0.70	9.79	± 0.36	9.80	± 2.84	7.80	± 1.71	17.70	± 1.31	10.79
	EX-BrdU + NeuN/DCX	14.75	± 10.64	14.34	± 1.48	16.10	± 2.47	12.16	± 1.40	11.12	± 0.82	10.06	± 1.32	14.63	± 0.87	8.62	± 0.87	18.78	± 2.16	12.96
	EX/SED %	85.43		129.95		151.85	∗	139.72		111.63		102.75		149.40	∗	110.52		106.13		121.57

## Supplementary Materials

Supplementary Materials Table 1: statistical data of proliferative NCSs in 9 mouse strains and the effects of exercise. A red box indicates a significant increase and a blue box indicates a significant reduction as compared to the cross-matched group. *p* value in the table shown compared to the cross-matched group obtained from LSD post hoc analysis. Between-subject effects *F* value and *p* value of mouse strain, exercise, and mouse strain and exercise (*n* = 5 per group). Table 2: statistical data of differentiating neuroblasts in 9 mouse strains and the effects of exercise. A red box indicates a significant increase and a blue box indicates a significant reduction as compared to the cross-matched group. *p* value in the table shown compared to the cross-matched group obtained from LSD post hoc analysis. Between-subject effects *F* value and *p* value of mouse strain, exercise, and mouse strain and exercise (*n* = 5 per group). Table 3: statistical data of integrated neurons in 9 mouse strains and the effects of exercise. A red box indicates a significant increase and a blue box indicates a significant reduction as compared to the cross-matched group. *p* value in the table shown compared to the cross-matched group obtained from LSD post hoc analysis. Between-subject effects *F* value and *p* value of mouse strain, exercise, and mouse strain and exercise (*n* = 5 per group).
