# Supplementary material for: Comparison of Adult Hippocampal Neurogenesis and Susceptibility to Treadmill Exercise in Nine Mouse Strains
Source: Neural Plast. 2017 Dec 17;2017:5863258. doi: 10.1155/2017/5863258 (PMC5748094; doi:10.1155/2017/5863258)
Supplement: Supplementary Materials — Table 1: statistical data of proliferative NCSs in 9 mouse strains and the effects of exercise. A red box indicates a significant increase and a blue box indicates a significant reduction as compared to the cross-matched group. p value in the table shown compared to the cross-matched group obtained from LSD post hoc analysis. Between-subject effects F value and p value of mouse strain, exercise, and mouse strain and exercise (n = 5 per group). Table 2: statistical data of differentiating neuroblasts in 9 mouse strains and the effects of exercise. A red box indicates a significant increase and a blue box indicates a significant reduction as compared to the cross-matched group. p value in the table shown compared to the cross-matched group obtained from LSD post hoc analysis. Between-subject effects F value and p value of mouse strain, exercise, and mouse strain and exercise (n = 5 per group). Table 3: statistical data of integrated neurons in 9 mouse strains and the effects of exercise. A red box indicates a significant increase and a blue box indicates a significant reduction as compared to the cross-matched group. p value in the table shown compared to the cross-matched group obtained from LSD post hoc analysis. Between-subject effects F value and p value of mouse strain, exercise, and mouse strain and exercise (n = 5 per group). [file 5863258.f1.docx]

| P-value |  | CB7BL/6J | | A/J | | BALB/c | | C3H/HeJ | | FVB | | 129/SvJ | | DBA/1 | | DBA/2 | | ICR | |
| --- | --- | --- | --- | --- | --- | --- | --- | --- | --- | --- | --- | --- | --- | --- | --- | --- | --- | --- | --- |
|  |  | SED | EX | SED | EX | SED | EX | SED | EX | SED | EX | SED | EX | SED | EX | SED | EX | SED | EX |
| CB7BL/6J | SED | - | .000 | .000 | .336 | .000 |  | .000 |  | .000 |  | .000 |  | .000 |  | .000 |  | .000 |  |
|  | EX |  | - | .000 | .000 |  | .000 |  | .000 |  | .000 |  | .000 |  | .000 |  | .000 |  | .000 |
| A/J | SED |  |  | - |  | .056 |  | .336 |  | .089 |  | .392 |  | .056 |  | .000 |  | .166 |  |
|  | EX |  |  |  | - |  | .003 |  | .336 |  | .007 |  | .830 |  | .004 |  | .000 |  | .285 |
| BALB/c | SED |  |  |  |  | - |  | .336 |  | .830 |  | .285 |  | 1.000 |  | .004 |  | .592 |  |
|  | EX |  |  |  |  |  | - |  | .035 |  | .748 |  | .001 |  | .915 |  | .000 |  | .044 |
| C3H/HeJ | SED |  |  |  |  |  |  | - |  | .453 |  | .915 |  | .336 |  | .000 |  | .668 |  |
|  | EX |  |  |  |  |  |  |  | - |  | .071 |  | .240 |  | .044 |  | .000 |  | .915 |
| FVB | SED |  |  |  |  |  |  |  |  | - |  | .392 |  | .830 |  | .002 |  | .748 |  |
|  | EX |  |  |  |  |  |  |  |  |  | - |  | .004 |  | .830 |  | .000 |  | .089 |
| 129/SvJ | SED |  |  |  |  |  |  |  |  |  |  | - |  | .285 |  | .000 |  | .592 |  |
|  | EX |  |  |  |  |  |  |  |  |  |  |  | - |  | .002 |  | .000 |  | .200 |
| DBA/1 | SED |  | Between-subjects effects F-value and p-value | | | | | |  |  |  |  |  | - |  | .004 |  | .592 |  |
|  | EX |  | Mouse strain | |  |  | F = | 43.363 | | p = | .000 | |  |  | - |  | .000 |  | .056 |
| DBA/2 | SED |  | Exercise | |  |  | F = | 100.311 | | p = | .000 | |  |  |  | - |  | .001 |  |
|  | EX |  | Mouse strain and exercise | | | | F = | 2.844 | | p = | .008 | |  |  |  |  | - |  | .000 |
| ICR | SED |  |  |  |  |  |  |  |  |  |  |  |  |  |  |  |  | - | .000 |
|  | EX |  |  |  |  |  |  |  |  |  |  |  |  |  |  |  |  |  | - |

**Supplementary Table. 1. Statistical data of proliferative NCSs in 9 mouse strains and the effects of exercise. A red box indicates a significant increase and a blue box indicates a significant reduction as compared to the cross-matched group. p-value in the table showed compared to the cross-matched group** **obtained from LSD post-hoc analysis.** **Between-subjects effects F-value and p-value of in mouse strain, exercise, and mouse strain and exercise.** (n = 5 per group).

| P-value |  | CB7BL/6J | | A/J | | BALB/c | | C3H/HeJ | | FVB | | 129/SvJ | | DBA/1 | | DBA/2 | | ICR | |
| --- | --- | --- | --- | --- | --- | --- | --- | --- | --- | --- | --- | --- | --- | --- | --- | --- | --- | --- | --- |
|  |  | SED | EX | SED | EX | SED | EX | SED | EX | SED | EX | SED | EX | SED | EX | SED | EX | SED | EX |
| CB7BL/6J | SED | - | .000 | .000 |  | .000 |  | .000 |  | .000 |  | .000 |  | .000 |  | .000 |  | .000 |  |
|  | EX |  | - |  | .000 |  | .000 |  | .000 |  | .000 |  | .000 |  | .000 |  | .000 |  | .000 |
| A/J | SED |  |  | - |  | .000 |  | .974 |  | .028 |  | .357 |  | .000 |  | .000 |  | .742 |  |
|  | EX |  |  |  | - |  | .000 |  | .063 |  | .000 |  | .003 |  | .000 |  | .000 |  | .078 |
| BALB/c | SED |  |  |  |  | - |  | .000 |  | .038 |  | .000 |  | .767 |  | .009 |  | .000 |  |
|  | EX |  |  |  |  |  | - |  | .000 |  | .025 |  | .000 |  | .818 |  | .000 |  | .000 |
| C3H/HeJ | SED |  |  |  |  |  |  | - |  | .030 |  | .341 |  | .000 |  | .000 |  | .767 |  |
|  | EX |  |  |  |  |  |  |  | - |  | .015 |  | .000 |  | .000 |  | .000 |  | .921 |
| FVB | SED |  |  |  |  |  |  |  |  | - |  | .002 |  | .018 |  | .000 |  | .059 |  |
|  | EX |  |  |  |  |  |  |  |  |  | - |  | .000 |  | .014 |  | .000 |  | .012 |
| 129/SvJ | SED |  |  |  |  |  |  |  |  |  |  | - |  | .000 |  | .000 |  | .213 |  |
|  | EX |  |  |  |  |  |  |  |  |  |  |  | - |  | .000 |  | .000 |  | .000 |
| DBA/1 | SED |  | Between-subjects effects F-value and p-value | | | | | |  |  |  |  |  | - |  | .020 |  | .000 |  |
|  | EX |  | Mouse strain | |  |  | F = | 149.893 | | p = | .000 | |  |  | - |  | .001 |  | .000 |
| DBA/2 | SED |  | Exercise | |  |  | F = | 272.811 | | p = | .000 | |  |  |  | - |  | .000 |  |
|  | EX |  | Mouse strain and exercise | | | | F = | 13.875 | | p = | .000 | |  |  |  |  | - |  | .000 |
| ICR | SED |  |  |  |  |  |  |  |  |  |  |  |  |  |  |  |  | - | .000 |
|  | EX |  |  |  |  |  |  |  |  |  |  |  |  |  |  |  |  |  | - |

**Supplementary Table. 2. Statistical data of differentiating neuroblasts in 9 mouse strains and the effects of exercise A red box indicates a significant increase and a blue box indicates a significant reduction as compared to the cross-matched group. p-value in the table showed compared to the cross-matched group** **obtained from LSD post-hoc analysis.** **Between-subjects effects F-value and p-value of in mouse strain, exercise, and mouse strain and exercise.** (n = 5 per group).

| P-value |  | CB7BL/6J | | A/J | | BALB/c | | C3H/HeJ | | FVB | | 129/SvJ | | DBA/1 | | DBA/2 | | ICR | |
| --- | --- | --- | --- | --- | --- | --- | --- | --- | --- | --- | --- | --- | --- | --- | --- | --- | --- | --- | --- |
|  |  | SED | EX | SED | EX | SED | EX | SED | EX | SED | EX | SED | EX | SED | EX | SED | EX | SED | EX |
| CB7BL/6J | SED | - | .000 | .000 |  | .000 |  | .000 |  | .000 |  | .000 |  | .000 |  | .000 |  | .000 |  |
|  | EX |  | - |  | .000 |  | .000 |  | .000 |  | .000 |  | .000 |  | .000 |  | .000 |  | .000 |
| A/J | SED |  |  | - |  | .071 |  | .332 |  | .226 |  | .903 |  | .031 |  | .001 |  | .009 |  |
|  | EX |  |  |  | - |  | .005 |  | .017 |  | .000 |  | .092 |  | .001 |  | .000 |  | .117 |
| BALB/c | SED |  |  |  |  | - |  | .396 |  | .544 |  | .092 |  | .715 |  | .092 |  | .000 |  |
|  | EX |  |  |  |  |  | - |  | .627 |  | .276 |  | .226 |  | .544 |  | .000 |  | .000 |
| C3H/HeJ | SED |  |  |  |  |  |  | - |  | .808 |  | .396 |  | .226 |  | .012 |  | .000 |  |
|  | EX |  |  |  |  |  |  |  | - |  | .117 |  | .466 |  | .276 |  | .000 |  | .000 |
| FVB | SED |  |  |  |  |  |  |  |  | - |  | .276 |  | .332 |  | .023 |  | .000 |  |
|  | EX |  |  |  |  |  |  |  |  |  | - |  | .023 |  | .627 |  | .003 |  | .000 |
| 129/SvJ | SED |  |  |  |  |  |  |  |  |  |  | - |  | .042 |  | .001 |  | .006 |  |
|  | EX |  |  |  |  |  |  |  |  |  |  |  | - |  | .071 |  | .000 |  | .002 |
| DBA/1 | SED |  | Between-subjects effects F-value and p-value | | | | | |  |  |  |  |  | - |  | .184 |  | .000 |  |
|  | EX |  | Mouse strain | |  |  | F = | 65.326 | | p = | .000 | |  |  | - |  | .001 |  | .000 |
| DBA/2 | SED |  | Exercise | |  |  | F = | 100.925 | | p = | .000 | |  |  |  | - |  | .000 |  |
|  | EX |  | Mouse strain and exercise | | | | F = | 2.018 | | p = | .056 | |  |  |  |  | - |  | .000 |
| ICR | SED |  |  |  |  |  |  |  |  |  |  |  |  |  |  |  |  | - | .001 |
|  | EX |  |  |  |  |  |  |  |  |  |  |  |  |  |  |  |  |  | - |

**Supplementary Table. 3. Statistical data of integrated neurons in 9 mouse strains and the effects of exercise. A red box indicates a significant increase and a blue box indicates a significant reduction as compared to the cross-matched group. P-value in the table showed compared to the cross-matched group** **obtained from LSD post-hoc analysis.** **Between-subjects effects F-value and p-value of in mouse strain, exercise, and mouse strain and exercise.** (n = 5 per group).
